# Molecular Resonance Imaging of the CAIX Expression in Mouse Mammary Adenocarcinoma Cells

**DOI:** 10.3390/ph16091301

**Published:** 2023-09-14

**Authors:** Claudia Quattrociocchi, Alberto Mangia, Silvio Aime, Valeria Menchise, Daniela Delli Castelli

**Affiliations:** 1 Department of Molecular Biotechnology and Health Sciences, Molecular Biotechnology Center, University of Turin, 10126 Turin, Italy; claudia.quattrociocchi@unito.it (C.Q.); alberto.mangia@unito.it (A.M.); 2 CNR (Consiglio Nazionale delle Ricerche), Institute of Biostructures and Bioimaging, Molecular Biotechnology Center, 10126 Turin, Italy; silvio.aime@unito.it (S.A.); valeria.menchise@unito.it (V.M.)

**Keywords:** MRI, molecular imaging, CAIX, breast cancer, Gd-contrast agent, liposomes

## Abstract

The carbonic anhydrase isoform IX (hCAIX) is one of the main players in extracellular tumor pH regulation, and it is known to be overexpressed in breast cancer and other common tumors. hCA IX supports the growth and survival of tumor cells, and its expression is correlated with metastasis and resistance to therapies, making it an interesting biomarker for diagnosis and therapy. The aim of this work deals with the development of an MRI imaging probe able to target the extracellular non-catalytic proteoglycan-like (PG) domain of CAIX. For this purpose, a specific nanoprobe, LIP_PepC, was designed by conjugating a peptidic interactor of the PG domain on the surface of a liposome loaded with Gd-bearing contrast agents. A Mouse Mammary Adenocarcinoma Cell Line (TS/A) was chosen as an in vitro breast cancer model to test the developed probe. MRI results showed a high selectivity and sensitivity of the imaging probe toward hCAI-expressing TS/A cells. This approach appears highly promising for the in vivo translation of a diagnostic procedure based on the targeting of hCA IX enzyme expression.

## 1. Introduction

Despite significant advances in therapy, breast cancer (BC) is still the second leading cause of cancer death in women [1]. This indicates that a better understanding of this multifaceted disease is needed. Recent years have witnessed a growing interest in pH deregulation phenomena taking place in both the intracellular and extracellular tumor environments. The study of H^+^ dynamics in cancer has led to a new paradigm known as the pH-centric anticancer paradigm. Such metabolic reprogramming involves the intracellular alkalinization of cancer cells associated with extracellular microenvironmental acidosis [2,3]. The alteration of the pH gradient (pHi to pHe) confers important advantages to cancer cells and tissues, such as the enhancement of their resistance to hypoxia and cancer therapy [4,5]. Among others, one of the main players in pH regulation in cancer cells is the transmembrane isoform of carbonic anhydrases (hCAs), hCAIX. This enzyme is a type I transmembrane dimeric protein consisting of an extracellular part containing the catalytic domain and a unique proteoglycan-like (PG) domain, a transmembrane (TM) region. and an intracellular (IC) tail. This protein is known to be overexpressed in breast cancer, as well as in many common tumors, and to play a critical role in hypoxia-associated tumor acidosis [6,7,8,9,10].

The presence of a targetable extracellular domain and the limited expression in healthy tissues have contributed to making CAIX an increasingly important biomarker for cancer diagnosis and an interesting site for the design of innovative therapies.

Indeed, many CAIX inhibitors have been developed and are currently under intense scrutiny in dedicated clinical trials [11].

Owing to its strategic role in pH regulation, CAIX has gained a crucial role as a diagnostic marker. Indeed, its expression level, measured after biopsy, can help distinguish between malignant and benign lesions in some solid tumors [12]. The possibility of visualizing the CAIX expression in vivo through non-invasive imaging techniques is a very appealing goal. An interesting imaging strategy has been reported by More et al., who developed a novel [F-18]-PET tracer based on acetazolamide, a well-known CA-IX inhibitor [13]. A related near-infrared (NIR) fluorescent dye targeting the CA IX has been proposed to visualize hypoxic regions in murine models. Its application in cancer image-guided surgeries has been discussed [14].

Although there are many studies investigating CAIX targeting using optical and nuclear imaging techniques, until now, few Magnetic Resonance Imaging (MRI) approaches have been reported [15]. MRI is a technique endowed with superior soft tissue contrast and excellent spatial resolution. MRI is less invasive than nuclear imaging due to non-radioactive contrast agents and can penetrate tissues deeply, unlike optical imaging.

Conversely, the sensitivity of MRI probes is rather low, making the ability to visualize a molecular marker in vivo strongly dependent on the target concentration and/or on the efficacy and the amount of the contrast agent accumulated at the site. To enhance the concentration of the contrast agent delivered at the target site, an established strategy consists of the use of paramagnetic liposomes that can transport a large amount of contrast agents per single vesicle, thus amplifying the effect of the molecular recognition event [16].

Liposomes are the most biocompatible clinically approved nano-sized delivery systems [17], entrapping an aqueous core inside a phospholipidic bilayer [18]. Multifunctionalized phospholipids can be considered for the membrane formulation to prepare versatile vesicles endowed with targeting properties [19].

The development of stimulus-sensitive nanovesicles has contributed to improving the in vivo performance of MRI-responsive agents [20]. One approach exploits the process of “quenching/de-quenching”, which consists of using “silenced” probes that recover their relaxation-enhancing property upon the changes in the liposomes’ membranes. These “T1-quenched” nanosystems are designed to recover the signal-enhancement ability following the release of the vesicle’s payload in response to biological events, such as pH change, liposome–protein interaction, and vesicle degradation, thus allowing a sensitive in vivo MRI detection.

This work aims to develop an MRI diagnostic approach to evaluate the level of expression of hCAIX in a breast cancer cell model. This objective is pursued by designing a novel Gd-based MRI nanoprobe, referred to as LIP_PepC, carrying a CAIX PG domain targeting vector on the surface and a “quenched/de-quenched” T1-contrast system able to report on the specific delivery of the cargo of MRI probes at the target site and on its successive cellular uptake.

## 2. Results

### 2.1. Probe Synthesis and Characterization

**Scheme 1:**
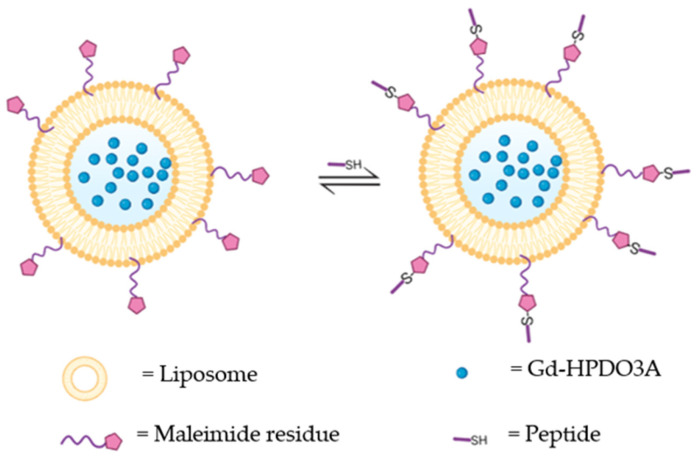
Liposome loaded with Gd-HPDO3A and bioconjugated with the targeting peptide.

The strategy of the probe design consists of functionalizing the surface of paramagnetic liposomes, loaded with Gd-HPDO3A, with a maleimide group that can be used as an anchor point for the peptide molecular vector directed toward CAIX (Scheme 1). This type of bioconjugation was chosen because it requires mild conditions, such as room temperature and pH 6.5, to prevent biomolecule degradation.

**Figure 1:**
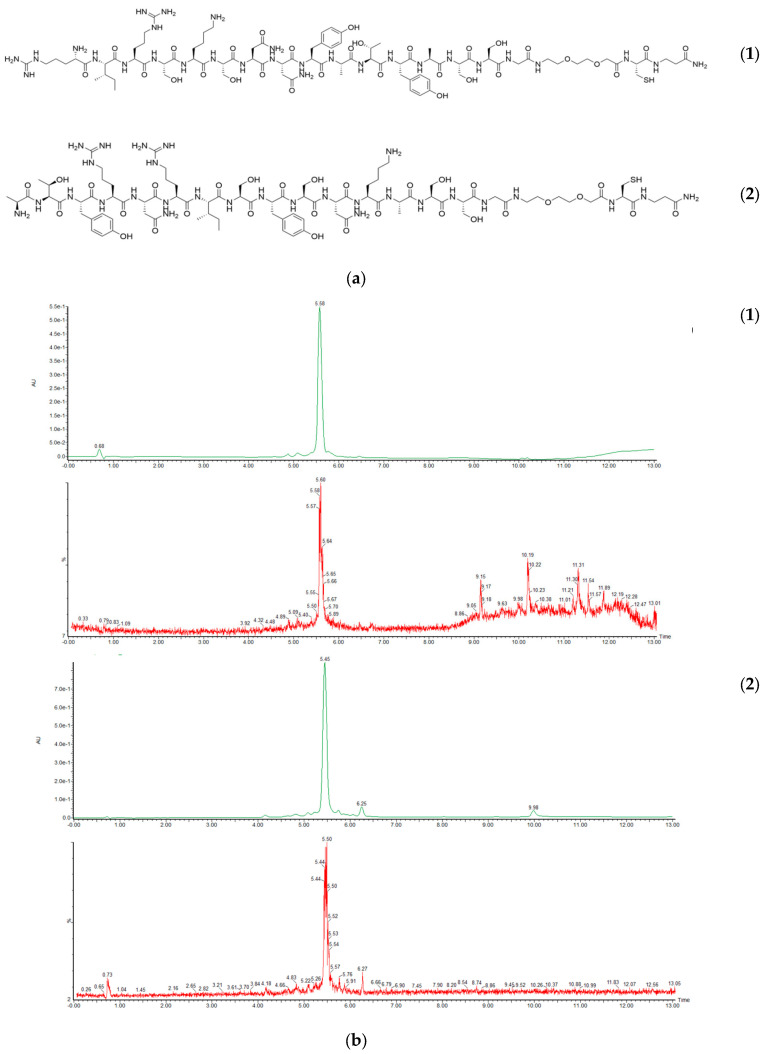
(**a**) Sequence of the targeting peptide (PepC) (**1**) and of the scramble peptide (SCR) (**2**); (**b**) post-purification chromatograms of PepC (**1**) and SCR (**2**).

First, we designed the amino acid sequence of the targeting peptide to be conjugated with the nano-sized probe. The sequence was designed considering the PG domain of hCAIX as the target since this region represents a unique feature of this enzyme that is absent in all the other hCAs. Recently, the crystallographic structure of the M75 antibody in complex with an epitope of the PG region [21] allowed the development of new peptides whose sequences reproduce the main Ab motifs involved in the intermolecular interaction. Extensive molecular dynamics simulations allowed us to optimize the affinity of the peptides toward the enzyme-targeting site. The most promising peptide developed in the study (Pep1) was chosen as a basis for the design of a peptide able to target the PG region of CAIX via its conjugation on the surface of a liposome. For this purpose, a cysteine residue was added to the Pep1 to introduce a free thiol group for further conjugation to a maleimide group that was properly incorporated into the nanoprobe external surface (see below). To improve the flexibility and the hydrophilicity of the molecule, an ASSG sequence and a PEG-based spacer were inserted before the cysteine residue. Figure 1a shows the sequence of the targeting peptide (PepC) and of the scrambled peptide (SCR) generated as a negative control. Both peptides were prepared through the automated solid-phase peptide synthesis (SPPS) technique [22]. The peptide’s crude purities were assessed upon ultra-performance liquid chromatography–mass spectrometry (UPLC-MS) analysis, and fractions with a purity higher than 90% were obtained through an automated liquid chromatography system (AKTA) (Figure 1a).

In order to prepare the paramagnetic liposomes, DPPC, DSPE-PEG 2000 MeO, and DSPE-PEG 2000 Mal at a 95:4:1 molar ratio were dissolved in chloroform and dried to form a thin lipidic film, which was hydrated with a 300 mM aqueous solution of the paramagnetic complex Gd-HPDO3A (ProHance). The mixture was sonicated until a suspension of liposomes with an average size of 120 nm was obtained, as measured through the dynamic light-scattering method. The final suspension was allowed to react at room temperature with a stoichiometric excess of PepC to functionalize all maleimide residues present on the liposomal surface. The concentration of Gd-HPDO3A in the suspensions, as determined by NMR magnetic susceptibility measurement, allowed us to estimate the concentration of the targeting liposome (LIP_PepC). A liposome functionalized with the scrambled peptide (LIP_SCR) was prepared following the same procedure. Also, the unfunctionalized control liposome (LIP) was synthetized. It contained DSPE-PEG 2000 in place of the DSPE-PEG 2000 Mal component.

The average size of the liposomes remained consistent with the measurement that occurred before the bioconjugation step. The size was shown to be about 120 nm for all three types of probes. The final concentration of Gd-HPDO3A in the liposome suspensions was determined through NMR magnetic susceptibility measurement (Evans assay) [23], and it was found to be 4.1 mM. The relaxometric measurements, at 20 MHz and 298 K, yielded, for LIP_PepC, r1p values of 0.12 mM^−1^ s^−1^ for the intact liposome and 4.7 mM^−1^ s^−1^ for the lyased liposome. For LIP_SCR, 0.13 mM^−1^ s^−1^ was found for the intact liposome. 4.7 mM^−1^ s^−1^ for the lyased liposome, 0.14 mM^−1^ s^−1^ for the intact liposome, and 4.7 mM^−1^ s^−1^ for the lyased liposome in the case of LIP. The determination of the total amount of Gd and the size of liposomes allowed us to assess a molar concentration of liposomes of 220 nM (Section 4).

### 2.2. MRI Tests on TS/A Cells

The Mouse Mammary Adenocarcinoma Cell Line (TS/A) was chosen as the in vitro breast cancer model to test the performance of the developed probe. This cell line is known to overexpress CAIX.

The labeling experiments were designed to assess the MRI Signal Enhancement (SE) in cellular pellets at time zero (i.e., immediately after the liposomes were added to the cell suspension) and 24 h later to allow liposomes’ uptake from the cells via CAIX-mediated internalization. The latter experiment is intended to assess the occurrence of the intracellular de-assembly of the liposomes, a necessary condition for detecting their MRI visualization.

**Figure 2:**
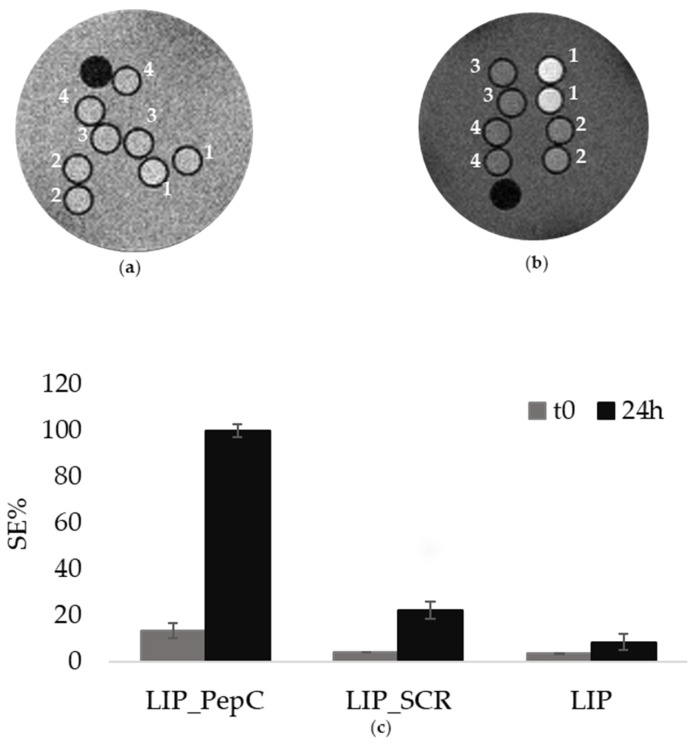
(**a**) MRI T1W of TS/A cells incubated with LIP_PepC (1), LIP_SCR (2), LIP (3), medium (4), and acquired on pellets obtained from the TS/A cells immediately after the addition of the liposomes to the cell culture. (t0); (**b**) MRI T1W of TS/A cells incubated with LIP_PepC (1), LIP_SCR (2), LIP (3), medium (4), and acquired after 24 h of incubation (24 h); (**c**) Histograms showing the SEs for Figure 2a,b.

The cells were incubated either with the specific targeting probe (LIP_PepC) or with the non-specific one containing the scrambled peptide (LIP_SCR), as well with the liposomes without any peptide on their surface (LIP), in order to take into account possible aspecific interactions. The final concentration of liposomes in the incubation medium was 56 nM. Cells were washed with PBS and removed from plates after 4 h of incubation at 37 °C. Cells were then transferred into 1 mm capillaries and centrifuged to obtain pellets for MRI analysis. A phantom containing cell capillaries, two for each set of experimental conditions and two for unlabeled cells, was prepared and imaged at 7T micro-imager. Figure 2 displays the signal enhancement (SE) for the eight capillaries acquired with the T1W sequence.

At time = 0, i.e., immediately after the addition of the liposomes to the cell suspension, no significant difference in the observed SEs was detected for the three specimens. (Figure 2a). This could be ascribed to the fact that the liposomes, even if bound to the CAIX exposed on the cell membrane, are still intact and hence almost silent. The experiment was then performed on specimens obtained from cells incubated at 37 °C for 24 h, after the unbound probe removal (Figure 2b). This additional time had the purpose of allowing liposomes to be eventually internalized and de-assembled. The results clearly showed that SEs for TS/A incubated 24 h after the probe removal were significantly larger than the ones observed at time 0. By assigning a value of 100% to the SE observed for cells incubated with LIP_PepC, the cells incubated with LIP_SCR or LIP were at 22% and 8%, respectively (Figure 2c). This indicates a very good specificity of the LIP_PepC towards TS/A cells and also shows that this specificity yields an excellent MRI response after 24 h of incubation, i.e., when the internalized probe has released the paramagnetic molecules into the cells. ICP mass analysis of the TS/A pellets yielded a value of 1.75 × 10^−15^ moles of Gd per cell, corresponding to a number of internalized liposomes per cell of 6000.

**Figure 3:**
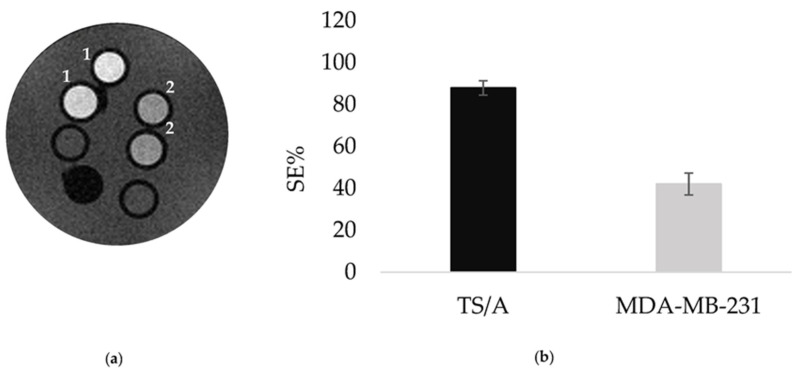
(**a**) MRI T1W of TS/A (1) and MDA-MB-231 (2) cells incubated with the same concentration of LIP_PepC and acquired after 24 h; (**b**) Histogram showing the SE referred to Figure 3a.

In order to ascertain that the specificity observed is due to CAIX targeting, a further experiment was performed comparing the SE of a specimen containing TS/A and MDA-MB-231 cells both incubated with LIP_PepC and imaged after 24 h post incubation at 37 °C. MDA-MB-231 cell lines are known for their lower expression level of CAIX [24]. The marked difference in the observed SEs for the two cell lines clearly supports the view of the correlation between CAIX expression and signal enhancements (Figure 3).

**Figure 4:**
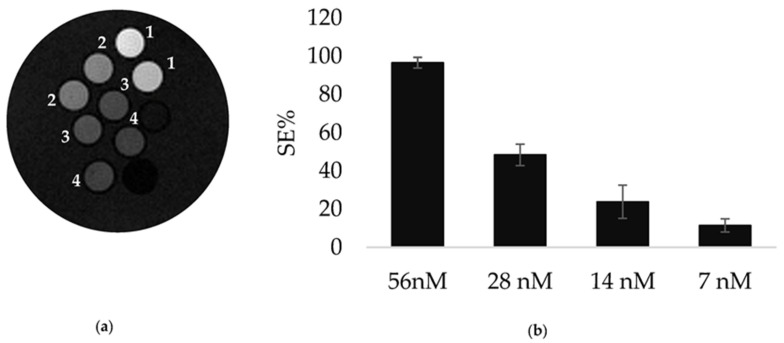
(**a**) MRI T1W of TS/A cells incubated with different concentrations of LIP_PepC: 56 nM (1), 28 nM (2), 14 nM (3), 7 nM (4); (**b**) histogram showing the SE for Figure 4a.

Next, TS/A cells have been incubated with decreasing concentration of LIP_PepC to test the sensitivity threshold of the targeting protocol. An enhancement of nearly 10% can still be observed for cells incubated with 7 mM of LIP_PepC (Figure 4).

## 3. Discussion

In most solid tumors, the metabolic changes necessary for the high rate of cancer cell proliferation result in alteration in intra- and extracellular pH. Tumor cells overexpress proteins that can expel protons, acidifying the extracellular environment (from 7.4 to 6.5–6.8) while preventing the decrease in intracellular pH that would cause cell apoptosis.

Overall, the central role played by carbonic anhydrases, in particular CAIX and CA XII, is now well established. These metalloenzymes catalyze the reversible transformation of CO_2_ into the bicarbonate ion with the release of a proton, thus modulating the concentration of the species required for cancer cell survival and proliferation. In recent years, carbonic anhydrases IX and XII have thus entered into the class of the most studied molecular targets in oncology [2,3,4,5,6,7,8,9,10,11]. Likewise, in the selection of an accessible target for in vivo imaging of the tumor microenvironment, carbonic anhydrase undoubtedly represents an ideal candidate. Furthermore, it has been recently demonstrated that in breast cancer, hCAIX and hCAXII exhibit distinct and non-overlapping expression patterns [25,26], where hCAXII has the high expression associated with better survival statistics, while hCAIX’s expression is associated with a more aggressive tumor phenotype, metastasis, and resistance to therapies. Alongside this aspect, an additional reason for our focus on CAIX is the presence in its structure of an extracellular, proteoglycan-like domain (PG), which is absent in all other forms of CAs, including CAXII. This unique structural feature of CAIX is becoming an increasingly attractive target in the development of CAIX inhibitory fragments. Indeed, the PG domain seems to play a decisive role in supporting the CA catalytic activity and is involved in cell–cell adhesion and intercellular communication, since both PG deletion and treatment with the PG-binding antibody M75 reduce adhesion and the spread of cancer cells [21,27]. Compared with the numerous high-affinity inhibitors of the CA catalytic site, inhibitors directed against the PG region are very promising, as they can provide high selectivity. Very recently, it was reported that the humanized antibody CA9hu-2 against CAIX PG domain showed a high antitumoral efficacy with the absence of cross-reaction with other CAs [28].

Similarly, the development of a diagnostic imaging protocol to visualize the expression of CAIX in vivo by distinguishing it from other isoforms would have a huge prognostic potential. The PG-targeting-based imaging techniques explored so far are predominantly optical imaging and PET/CT, both displaying high sensitivity and thus a remarkable ability to visualize even low concentrations of targets in vivo. The authors of [29] reported on the use of a radiolabeled antibody, G250, for PET-CT imaging of advanced renal cancer. This clinical application showed that it was possible to visualize tumor areas with high CAIX expression, as confirmed by immunohistochemistry data.

The low availability of MRI studies is undoubtedly due to the sensitivity of the technique, which is inadequate to detect the in vivo concentrations characteristic of this type of molecular target. On the other hand, the development of MRI imaging procedures would be strongly desirable given the low invasiveness of the technique, combined with a high resolution, which allows the acquisition of detailed morphological images. The strategy proposed in our study aims to overcome these limitations by using liposomal probes capable of (i) carrying a large amount of contrast agent per single target and (ii) being up-taken into the cells with the consequent release of their content.

It is well established that entrapping a large amount of paramagnetic molecule in the core of a liposome results in a “quenching” of their T1 effect on the proton relaxation rate of bulk water. This happens because the exchange rate between the water inside and outside the liposome is lower than the relaxation rate of compartmentalized water protons. Therefore, the release of liposome payload (or the extensive disruption of the membrane structure) results in the “dequenching” of the relaxation effect, which in turn produces an increase in the MRI signal. In several studies, the “quenching” of paramagnetic agents embedded in liposome’s inner cavity has been exploited to develop probes responsive to the release of liposome contents at the site of interest [30,31]. The rationale for choosing the LIP_PepC system, loaded with a “quenched” Gd-based contrast agent solution, for imaging CAIX expression lies in the possibility of following the fate of the probe once it reaches its destination.

In principle, the MRI approach allows important insights to be obtained about the process associated with the binding interaction between LIP_PepC and the CAIX PG domain. First, the probe could remain linked to the outer cell membrane surface, where it gives rise to a low MRI signal. Next, the liposome may degrade still in the extracellular space, and this process is expected to produce a limited SE owing to the release of Gd-complex that would be quickly washed away. In another scenario, the probe could be internalized, and the paramagnetic vesicle could remain intact in the intracellular medium (low SE). Finally, as shown in this study with the TS/A cells, it could be degraded intracellularly with a strong SE effect.

In fact, the analysis of the MR images acquired within a 24 h timeframe following the end of the incubation of TS/A and MDA-MB-231 cells with LIP_PepC and non-targeting liposomes revealed a marked increase in the SE only for TS/A incubated with LIP_PepC. These results undoubtedly demonstrated that there is a specificity toward CAIX and that the binding between the probe and the enzyme is able to induce receptor-mediated internalization, as also reported for an antibody directed against the active site of CAIX [28]. In particular, the progressive increase in signal enhancement, starting from a barely detectable contrast at t = 0 and ending with a final enhancement of 100% after 24 h allows us to propose a receptor-mediated internalization mechanism, after which the probe gradually releases the contrast agent, which accumulates in the intracellular medium, reducing the T1 of intracellular water over time. Assuming this CAIX-mediated internalization process, the low SE observed at t = 0 would correspond to the integer and “quenched” probe, while the increase in the MRI signal over time indicates that the paramagnetic solution is gradually escaping somehow from the liposomal core to the cytosol. Furthermore, the concentration of Gd per cell measured using ICP enables us to calculate approximatively the number of liposomes internalized per cell, estimated to be about 6000.

The evidence presented in this study regarding the internalization of the probe and the subsequent release of its contents could pave the way for the advancement of novel therapeutic approaches in anticancer drug delivery.

## 4. Materials and Methods

### 4.1. Solid Phase Peptide Synthesis

All Fmoc-protected amino acids were purchased from Novabiochem^®^, and N,N-diisopropylcarbodiimide (DIC), OxymaPure^®^, and Fmoc-H-Rink Amide ChemMatrix resin (0.4 mmol/g) were purchased from Sigma-Aldrich Co. (Merck, St. Louis, MO, USA). Solvents for synthesis, deprotection reagents, and cleavage reagents were of synthesis grade and purchased from Sigma-Aldrich Co. (Merck, St. Louis, MO, USA). Solvents and other chemicals used for high-performance liquid chromatography (HPLC) were purchased from VWR International. Peptide synthesis sequences were synthesized using a Liberty Blue™ automated microwave peptide synthesizer purchased from CEM (Matthews, NC, USA), following a standard Fmoc protocol. The H-Rink Amide ChemMatrix resin was used as the solid support. Standard couplings of amino acids were carried out in DMF using DIC/OxymaPure^®^ activation and piperidine deprotection. After completing synthesis, the resin with the bound peptide was moved into a reactor for washings and then cleavage. Washings were performed with 100 mL of Dimethylformamide (DMF) first, then with 100 mL of dimethyl carbonate (DMC), and finally with 25 mL × 2 of EtOEt. For drying, the reactor was connected to the vacuum pump for 1 h. The peptide was cleaved from the resin manually by using Trifluoroacetic acid (TFA) under gentle agitation for 2 h at room temperature in the presence of scavengers (standard cleavage solution: TFA/Phenol/MilliQWater/Triisopropyl silane (TIPS) 88:5:5:2) to avoid oxidation. After filtration, the crude peptides were precipitated by the addition of cold diethyl ether, centrifuged, washed with cold Et2O five times, dried, and dissolved in ultrapure water.

Analyses of synthesized peptides were carried out by UPLC-MS Acquity H-Class Plus on an Acquity UPLC Peptide BEH C18 column (300 Å, 1.7 µm, 2.1 mm × 100 mm), both purchased from Waters, with solvent B (acetonitrile with 0.05% TFA) versus solvent A (water with 0.05% TFA) using a 5–95% to a 100–0% gradient, at a flow rate of 0.4 mL/min for 13 min. Purification was performed through an HPLC AKTA purifier 10 on an X Terra Perp MS C18 OBDTM (5 µm, 19 mm × 100 mm) column in order to recover a fraction that would ensure the highest purity, and a new UPLC-MS analysis was performed. Finally, peptides were frozen, lyophilized, and stored at −20 °C.

### 4.2. Liposomes

Liposomes were prepared using the thin-film hydration method [32]. The lipid mixture chosen for liposome synthesis comprised DPPC (1, 2-Dipalmitoyl-sn-glycero-3-phosphocholine), DSPE-PEG 2000 MeO (1,2-Distearoyl-sn-glycero-3-phosphoethanolamine-N-[methoxy(polyethyleneglycol)-2000], ammonium salt), and DSPE-PEG 2000 Mal (1,2-distearoyl-sn-glycero-3-phosphoethanolamine-N-[maleimide(polyethylene glycol)-2000] ammonium salt) at a 95:4:1 molar ratio. All lipids were purchased from Sigma-Aldrich Co. (Merck, St. Louis, MO, USA). The total amount of phospholipids was 20 mg/mL, dissolved in chloroform, which was slowly evaporated until a thin film was formed. The lipidic film was left under vacuum until the total evaporation of chloroform (2 h) and then hydrated with a 300 mM aqueous solution (pH = 6.7) of the paramagnetic complex Gd-HPDO3A (ProHance). Gd-HPDO3A (HPDO3A = 1,4,7-tris(carboxymethyl)-10-(20 -hydroxypropyl)-1,4,7,10-tetraaza-cyclododecane) is commercially available from Bracco Imaging S.p.A under the trade name ProHance. To generate liposomes, the mixtures were sonicated using an immersion-tip instrument (SONOPULS HD 2070 Bandelin ultrasonic homogenizer, 20 KHz; Bandelin Electronic, Berlin, Germany). Sonication was performed in 2 cycles of 45 s each, with 30% power. The suspension was reacted with a controlled excess of synthesized peptides to functionalize all maleimide residues present on the liposomal surface. The final suspension was purified from a non-encapsulated metal complex and unbound peptide by dialysis carried out against an isotonic HEPES buffer (5 mM HEPES, 0.15 M NaCl, pH 7.4) at 4 °C using a dialysis cellulose membrane with 14 KDa molecular weight cut-off purchased from Sigma-Aldrich, St. Louis, MO, USA. After purification, the liposome size, the mean diameter, and the polydispersity index (PDI) were determined using dynamic light scattering (DLS, Zetasizer Nano ZS, Malvern, UK) at 25 °C and an angle of 90° upon diluting the liposome solution 1:100 in HEPES/NaCl buffer. The final concentration of Gd-HPDO3A in the liposome suspensions was determined using NMR magnetic susceptibility measurement, Evans assay, with a Bruker 600 MHz spectrometer (AVANCE 600, 14 T). The total amount of Gd and the size of the liposomes allowed the molar concentration of the liposomes to be determined using the following formula:

$$\left[Lip\right]M=\frac{\frac{{\left[Gd\right]}_{tot}}{{Vol}_{lip}\times {\left[Gd\right]}_{hydr}}}{N_{A}}$$

where [*Gd*]*_tot_* is the concentration of Gadolinium in the overall liposomes suspension, [*Gd*]*_hydr_* is the concentration of Gadolinium in the hydration solution, used for the liposome preparation, *Vol_lip_* is the volume of liposomes (in L), and *N_A_* is the Avogadro’s number.

### 4.3. Cell Lines and Incubation Protocol

TS/A cells (kindly provided by Prof. F. Cavallo’s group, University of Turin) were cultured in L-GLUT RPMI 1640 (Euroclone) medium, supplemented with 10% (*v*/*v*) fetal bovine serum (FBS, Sigma-Aldrich, St. Louis, MO, USA) and 1% Penicillin-Streptomycin (10,000 IU/mL penicillin, 10,000 IU/mL streptomycin). MDA-MB-231 cells (kindly provided by Prof. D. Longo’s group, University of Turin) were cultured in DMEM high-glucose (Euroclone) medium, supplemented with 10% (*v*/*v*) fetal bovine serum (FBS, Sigma-Aldrich, St. Louis, MO, USA), 1% (*v*/*v*) penicillin–streptomycin (10,000 IU/mL penicillin, 10,000 IU/mL streptomycin) and, if not present, Glutamine 4 nM. These cells were maintained in a 5% CO_2_ incubator at 37 °C until the cells attained 80% confluency and were further used for in vitro experiments. For the cell-binding experiments, 1 × 10^6^ cells were seeded in 6 cm diameter dishes. After 24 h, the cells were incubated with the binding solution consisting, of a medium solution with a liposome concentration of 56 nM, for 4 h at 37 °C and 5% CO_2_. At the end of the incubation, cells were washed three times with 2 mL of PBS and detached using a cell scraper. Cells were also transferred into glass capillaries for MRI analysis.

### 4.4. MRI Analysis

Cells were placed in glass capillaries inside an agar phantom, and MR images were acquired using a standard T1-weighted multi-slice spin echo sequence (TR (repetition time)/TE (echo time)/NEX (number of excitations) = 150/3.0/8, FOV (field of view) = 10 × 10 mm, slice thickness = 1 mm, matrix size 128 × 128) on a Bruker Avance300 spectrometer (7T) equipped with a Micro 2.5 microimaging probe (Bruker BioSpin, Ettlingen, Germany).

The Mean Signal Intensity (SI) was determined on T1W images by manually drawing regions of interest (ROIs) inside the capillaries with ImageJ 1.53t software (http://rsb.info.nih.gov/ij/). The T1 contrast enhancement (T1enh%) was calculated using the following formula:

$$SE\%=\frac{{SI}_{sample}-{SI}_{ref}}{{SI}_{ref}}\times 100$$

where *SI_sample_* and *SI_ref_* are signal intensities of cells incubated with the binding solution and pure medium, respectively. Each value was normalized with respect to the noise signal.

## 5. Conclusions

In summary, this study has demonstrated that the probe LIP_PepC shows high specificity for the CA-IX isoform with a good sensitivity threshold. These findings provide a solid basis for the preclinical translation of CAIX PG-domain-targeting probes in murine models. As a future perspective, we would like to extend this approach to target the CAXII isoform. This would allow the design of peptidic nano-vectors that selectively target either hCAIX or hCAXII isoforms in vivo, both of which are correlated with different prognostic severities in breast cancer. The determination of the expression ratio between hCAIX and hCAXII could potentially lead to the identification of a new biomarker for tumor proliferation.

## Data Availability

Data is contained within the article.

## Abbreviations

hCAIX, Carbonic Anhydrase isoform IX; PG, proteoglycan-like domain; TS/A, Mouse Mammary Adenocarcinoma Cell Line; MRI, Magnetic Resonance Imaging; PepC, targeting peptide; SCR, scrambled peptide; ProHance, Gd-HPDO3A; LIP_PepC, liposome functionalized with the CAIX targeting peptide; LIP_SCR, liposome functionalized with the scrambled peptide; LIP; control liposome without peptide.
